# The role of fatty acids in insulin resistance

**DOI:** 10.1186/s12944-015-0123-1

**Published:** 2015-09-29

**Authors:** Barry Sears, Mary Perry

**Affiliations:** Inflammation Research Foundation, 200 Corporate Place, Peabody, MA 01960 USA

**Keywords:** Insulin resistance, Inflammation, Fatty acids, Palmitic acid, Omega-3 fatty acids, Hypothalamus, Adipose tissue, Liver, Muscle, Endotoxemia

## Abstract

Insulin resistance is a multi-faceted disruption of the communication between insulin and the interior of a target cell. The underlying cause of insulin resistance appears to be inflammation that can either be increased or decreased by the fatty acid composition of the diet. However, the molecular basis for insulin resistance can be quite different in various organs. This review deals with various types of inflammatory inputs mediated by fatty acids, which affect the extent of insulin resistance in various organs.

## Introduction

The human body has developed an extraordinary number of systems to maintain stable blood glucose and to avoid broad swings in its level. These systems include hormones that are directly or indirectly generated by the diet. These hormones sense dietary nutrients and send appropriate neural signals to the brain (specifically the hypothalamus) to orchestrate fuel usage for either oxidation into energy or long-term storage. The central hormone involved in this metabolic communication system is insulin. However, increased inflammation can disturb these complex communication systems eventually leading to metabolic defects (obesity, metabolic syndrome, and diabetes).

Insulin is the primary regulator of carbohydrate, fat, and protein metabolism [1–3]. It inhibits lipolysis of stored fat in the adipose tissue and gluconeogenesis in the liver, it stimulates the translocation of the GLUT-4 protein to bring glucose into the muscle cells along with gene expression of proteins required for the optimal cellular function, cellular repair, and growth, and it indicates the metabolic availability of various fuels to the brain. Therefore keeping insulin within a therapeutic zone is critical for our survival.

In the past, access to adequate nutrients was a major concern. Today we have a new concern: Excess nutrient intake. However, even in this regard, insulin plays a primary role in defending the body against potential damage by using the adipose tissue, liver, and skeletal muscle as biological buffers against excess nutrient intake. This is important since all dietary nutrients are naturally inflammatory since their metabolism into other biological materials or conversion to energy can generate molecular responses that can activate increased inflammation [4]. This means that the intake of excess nutrients sets the foundation for the generation of excess inflammation. In the face of increased inflammation, the ability of insulin to orchestrate metabolism becomes compromised.

Obesity is different than insulin resistance. Obesity is defined as the excess of body fat. That itself is not necessarily an adverse condition as long as the fat is safely stored in healthy fat cells that respond to insulin. Insulin resistance is a condition in which cells are no longer responding appropriately to circulating insulin. Although there are many potential molecular causes of insulin resistance, ultimately they are all either directly or indirectly caused by increased inflammation.

### Insulin resistance

The definition of insulin resistance is deceptively simple (a condition in which cells are no longer responding appropriately to circulating insulin). Although the molecular mechanism is not fully understood, at the cellular level the strength of insulin signaling from its receptor to its final action is attenuated. In particular, if insulin receptor substrate-1 (IRS-1) is phosphorylated at a critical serine/threonine positions, this will lead to an accelerated degradation of the phosphorylated IRS-1 protein thereby reducing the strength of the insulin signaling [5, 6].

It is also known that certain short-term dietary changes can rapidly reduce insulin resistance before any significant fat loss occurs. This would include stringent calorie restriction to reduce insulin resistance within a matter of days [7]. Likewise, certain drugs, such as corticosteroids, can rapidly increase insulin resistance [8].

Furthermore there are various metabolic adaptations to stressors that can induce insulin resistance. These stressors include pregnancy, hibernation, and sepsis [1]. The increase in insulin resistance in response to these stressors is a method of diverting stored nutrients to address the necessary metabolic adaptation. Likewise sleep deprivation is another effective way of increasing insulin resistance in the short-term [9].

### The role of inflammation in insulin resistance

However, it is chronic insulin resistance that appears to be directly or indirectly related to diet-induced inflammation. The mechanisms at the molecular level are complex and manifold. They are based on the ability of increased cellular inflammation to interrupt insulin’s action by disrupting signaling mechanisms within the cell in particular by the enhancing the phosphorylation of IRS.

The primary suspects appear to be inflammatory mediators including the inflammatory cytokine tumor necrosis factor alpha (TNFα) as well as inflammatory protein kinases such as c-JUN N-terminal kinase (JNK) and the IKK complex [10].

TNFα knock-out animal models are resistant to the development of insulin resistance in animal strains prone to diet-induced obesity (DIO mice) or those that lack leptin (Ob/Ob mice) [11]. The JNK pathway is stress-activated and is associated with the presence of M1 activated macrophages [12]. If the IKK complex is activated by inflammation, it phosphorylates IκB (the inhibitor of NF-κB) leading to its rapid degradation. Once IκB is degraded, it can no longer prevent the free entry of NF-κB into the nucleus. Once NF-κB enters the nucleus it causes the expression of additional inflammatory mediators such as cytokines (IL-1, IL-6, TNFα, etc.) and enzymes such as COX-2 [13].

The suggestion that inflammation may be related to insulin resistance came more than a century ago when it was observed that certain anti-inflammatory drugs (salicylates and aspirin) were effective in reducing the hyperglycemia observed in diabetes [14–17]. It is now known that these drugs are inhibitors of phosphorylation action of the IKK complex [18, 19].

Table 1 summarizes the various inflammatory pathways, but the underlying general mechanism of each ultimately appears to be induced through increased inflammation within the cell.

**Table 1:** Potential inflammatory pathways leading to increased insulin resistance

TNFα	
JNK	
IKK	
Fatty acid-mediated effects	

The first three pathways have been discussed extensively in the literature; therefore this review will focus on the latter pathway.

Additional molecular mechanisms of insulin resistance include the lipid- overload hypothesis in which there is a build-up of diacylglycerides (DAG) or ceramides that inhibit the signaling of insulin as well as endoplasmic reticulum (ER) stress (induced by excess calories) or oxidative stress (induced by the generation of excess free radicals) [20–22]. Making these diverse molecular mechanisms of insulin resistance even more complex is that they are operative in some organs and not in others.

### Fatty acid-mediated insulin resistance in different organs

#### Overview

Insulin resistance can be characterized as a metabolic dysfunction that is often mediated by increased inflammation. Much of that inflammation may be diet-induced via the role of various dietary fatty acids. In particular, omega-6 and saturated fatty acids (especially arachidonic acid (AA) and palmitic acid) can be viewed as pro-inflammatory molecules, whereas omega-3 fatty acids (especially eicosapentaenoic acid (EPA) and docosahexaenoic acid (DHA)) can be viewed as anti-inflammatory molecules. This is because they have the ability to function as the necessary substrates to generate resolvins as well as binding to specific binding proteins that can decrease insulin resistance in an organ.

**Fig. 1:**
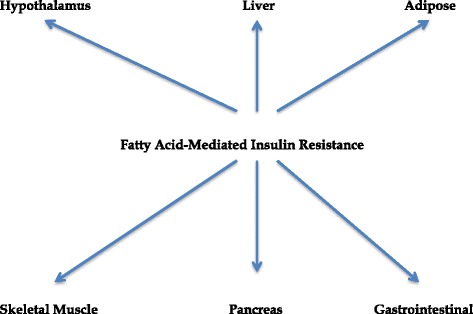
Organs affected by fatty acid-mediated insulin resistance

The various organs that can be affected by these fatty acid-mediated effects are shown in Fig. 1.

#### Hypothalamus

In many ways insulin resistance appears to start in the hypothalamus. The hypothalamus acts to match energy intake to energy expenditure to prevent excess accumulation of stored energy [23]. In particular, satiety signals from the gut are matched to adiposity (primarily-leptin) and blood (primarily-insulin) hormonal signals to control food intake [24, 25]. Unfortunately, either excess calories or saturated fats (especially palmitic acid) can cause inflammation in the hypothalamus, leading to resistance to the satiety signaling of both insulin and leptin [26–28]. As a result, satiety is attenuated and hunger increases. The hypothalamus also contains GPR120 binding proteins that are specific for long-chain omega-3 fatty acids such as EPA and DHA [29]. Thus the presence of adequate levels of these omega-3 fatty acids in the diet can decrease inflammation within the hypothalamus [30]. In fact, intracerebroventricular (icv) injections of omega-3 fatty acids into obese rats decrease insulin resistance [29–31]. Likewise, similar icv injections of anti-TLR-4 and anti-TNFα antibodies also decrease insulin resistance [32].

High-fat diets (HFD), especially those rich in saturated fats, are the standard method to cause diet-induced obesity in animal models. Increased inflammation appears in the hypothalamus within 24 h after beginning a HFD as indicated by increases in JNK and IKK proteins as well as increased expression of TLR-4 receptors and detection of ER stress [33]. IKK induces inflammation via activation of NF-κB, which inhibits the normal hormonal signaling of leptin and insulin necessary to create satiety. Activation of JNK is often preceded by the increase in ER stress [34]. This sets up a vicious cycle of increased hunger that eventually leads to the accumulation of excess calories as stored fat in the adipose tissue. It should be noted that the inflammation in the hypothalamus precedes any weight gain in the adipose tissue [35]. This also explains why significant calorie restriction can reduce insulin resistance before any significant loss in excess body fat in the adipose tissue. These experimental observations suggest that the hypothalamus is the central control point for the development of insulin resistance.

Excess nutrient intake (especially saturated fat) can also indirectly cause inflammation in the hypothalamus by activation of the TLR-4 receptors in the microglia in the brain eventually causing inflammatory damage to neurons in the hypothalamus [28]. It has been shown that with an extended use of a HFD that there is a decrease in the number of neurons responsible for generating satiety signals in the hypothalamus [36].

HFD diets are also associated with increased production of palmitic acid-enriched ceramides in the hypothalamus. This would provide still another link to the increased insulin and leptin resistance giving rise to increased hunger as satiety depends on functioning insulin pathways in the hypothalamic neurons [37].

Besides the presence of the GPR120 receptors in the hypothalamus, which if activated by omega-3 fatty acids decrease inflammation [38, 39], there are other fatty-acid-nutrient sensors in the hypothalamus that can be activated to increase inflammation. In particular, any increase in the free fatty-acid (FFA) levels in the blood can be sensed by the CD36/FATP-1 transporter at the surface of blood–brain-barrier (BBB). If those fatty acids are rich in palmitic acid (the primary product of *de novo* lipid production in the liver caused by excess dietary glucose), then the HPA axis is activated to release more cortisol thereby increasing insulin resistance [40]. On the other hand, if the fatty acid being sensed is primarily oleic acid, there will be a reduction in NPY (a powerful appetite-inducing hormone) expression in the hypothalamus that promotes satiety [41].

Finally there is the interaction of the hypothalamus with the liver via signaling through the vagus nerve [42]. This may explain why any inhibition of TNFα or TLR-4 signaling in the hypothalamus also decreases glucose production in the liver.

As you can begin to appreciate, the central regulation of appetite control by the hypothalamus is a very complex orchestration of the levels of inflammation and nutrient intake generated by the diet and the sensing of those levels by the hypothalamus.

#### Adipose tissue

We often think of obesity as the cause of insulin resistance, yet as described above, the genesis of insulin resistance appears to start in the hypothalamus with a disruption in the normal balance of hunger and satiety signals. As hunger increases, so does calorie intake.

The most effective site for storage of excess fat calories is the adipose tissue including those excess calories from carbohydrates that are converted to fat in the liver. The fat cells of the adipose tissue are the only cells in the body that are designed to safely contain large amounts of fat. This is why the adipose tissue is extremely rich in stem cells that can be converted to new fat cells to contain large levels of excess energy as triglycerides [43]. As long as those fat cells are healthy, there are no adverse metabolic effects (except excess weight) for the person. This is why approximately one-third of obese individuals fall into the category of “metabolically healthy obese” [44]. They have excess body fat but no metabolic disturbances that characterize the manifestation of insulin resistance.

However, fat cells do not have an unlimited capacity to expand. Even though the adipose tissue is highly vascularized, the over-expansion of existing fat cells can create hypoxia, which activates the HIF-1 gene [45, 46]. This results in the increased expression of both JNK and IKK thereby creating inflammation within the fat cell [47]. This inflammation, in turn, creates insulin resistance within the fat cell.

In the adipose tissue, insulin is normally an anti-lipolytic hormone as it decreases the activity of hormone-sensitive lipase (HSL), which is required to release stored fatty acids [48]. With the development of cellular inflammation and insulin resistance in the fat cell, higher levels of free fatty acids (FFA) can leave the fat cell to enter into the circulation and be taken up by other organs, such as the liver and the skeletal muscles that are unable to safely store large amounts of fat. As described later, this leads to developing insulin resistance in these organs. With increased inflammation in the fat cells, there is also a migration of greater numbers of M1 macrophages into the adipose tissue with a corresponding release of inflammatory cytokines, such as TNFα, which further increases insulin resistance and lipolysis [49, 50]. In the lean individual, only about 10 % of the adipose tissue mass is composed of macrophages, and those macrophages are primarily in the anti-inflammatory M2 state [51, 52]. In the obese individual up to 50 % of the mass of the adipose tissue may contain macrophages but now in the activated pro-inflammatory M1 state [51, 52]. Theoretically, new healthy fat cells could be generated from stem cells within the adipose tissue. However, that process requires the activation of the gene-transcription factor PPARγ [53]. The activity of this gene-transcription factor is inhibited by inflammatory cytokines, such as TNFα [54]. On the other hand, the activity of PPARγ is increased in the presence of anti-inflammatory nutrients, such as omega-3 fatty acids and polyphenols [55, 56]. Without the ability to form new healthy fat cells, the continued expansion of the existing fat cells eventually leads to cell death and further adipose tissue inflammation caused by incoming neutrophils and macrophages to clean the cellular debris caused by the necrotic fat cells [57].

As stated earlier, insulin resistance can inhibit the action of HSL due to increased hyperinsulinemia. Ironically, the increased hyperinsulinemia activates the lipoprotein lipase at the surface of the fat cell that hydrolyzes lipoprotein triglycerides to release free fatty acids [58, 59]. This also increases the synthesis of fatty-acids-binding proteins that bring the newly released FFA from the lipoproteins into the fat cells for deposition [60, 61]. The increase in fatty acid flux into the fat cells also requires greater synthesis of the FFA into triglycerides, but this can lead to ER stress activating the JNK pathway, thus further increasing insulin resistance in the fat cells [62]. This sets up a vicious cycle in which insulin resistance results in greater hunger (via insulin resistance in the hypothalamus) with increasing flux of FFA both into and out of the adipose tissue [63]. The cytokines being released by the pro-inflammatory M1 macrophages being attracted to the adipose tissue due to increasing cellular inflammation only increase this process by accelerating insulin resistance in the fat cells. This is why obese individuals with insulin resistance have greater levels of both the uptake and release of FFA into and from the adipose tissue. The increase in lipid influx causes an over-load of the synthetic capacity to make triglycerides, and as a result both DAG and ceramide levels begin to increase, which only further increases insulin resistance in the fat cells [64].

The speed of the inflammatory changes in the adipose tissue is not as rapid as they are in the hypothalamus. Whereas inflammatory changes can be seen in the hypothalamus within 24 h after beginning a HFD in animal models, it often takes 12–14 weeks to see similar changes in inflammation in the adipose tissue [65].

If the fat cells cannot expand rapidly enough to store this increasing fatty acid flow, then the excess released fatty acids begin to accumulate in other tissues such as the liver and skeletal muscles, and this begins the process of lipotoxicity that further increases systemic insulin resistance [66]. It is with the development of lipotoxicity that the real metabolic consequences of insulin resistance begin.

#### Liver

The liver can be viewed as the central manufacturing plant in the body. Raw materials (primarily carbohydrates and fats) are bought into the body to be processed by the liver and either stored (as liver glycogen) or repackaged as newly formed triglycerides (in the form of lipoproteins). The liver helps maintain stable glucose levels between meals by balancing glycogenesis (glycogen formation) and glycolysis of stored glycogen [67]. It should be pointed out that the glycogen stored in muscles can only be used internally as a source of energy and can’t be released back into the circulation to help maintain stable blood glucose levels.

Unlike the adipose tissue that can safely store excess fat, the liver cannot. Therefore of the first adverse metabolic consequences of insulin resistance is the build-up of fatty deposits in the liver. This is known as non-alcoholic fatty liver disease or NAFLD. Currently 20–30 % of Americans have NAFLD and 90 % of obese type-2 diabetic patients have NAFLD [68]. Ominously, it is estimated that 50 % of all Americans will have NAFLD by 2030 [67].

Another difference between the liver and the adipose tissue is the lack of infiltrating macrophages. Whereas a significant increase is observed in the levels of macrophages in the adipose tissue upon inflammation, it is the internal macrophages (Kupfer cells) in the liver that become activated. These activated Kupfer cells can now release cytokines that will further activate NF-κB in the liver cells.

Like hypothalamic inflammation, NAFLD can be rapidly generated in animal models within 3 days of starting a HFD [69]. This may be due to the direct linkage of the hypothalamus to the liver via the vagal nerve [70]. Once NAFLD is established, the ability of insulin to suppress liver glucose production is diminished without changes in weight, fat mass, or the appearance of any indication of insulin resistance in the skeletal muscle [71].

Because of the rapid build-up of fatty acids in the liver, the ability to convert them to triglycerides is also overwhelmed and DAG formation in liver increases [67, 71]. This is why the levels of DAG in the liver are the best clinical marker that chronic insulin resistance has begun to develop in that organ. The primary source of the fatty acids coming to the liver is via the adipose tissue because as the adipose tissue develops insulin resistance, the increased flow of FFA from the fat cells into the blood and therefore into the liver increases [72]. *De novo* lipid synthesis of fats from glucose in the liver is a smaller contributor to this increased flux of FFA into the liver [73]. Furthermore, liver insulin resistance is related only to the fatty acid levels in the liver, not the levels of visceral fat [74]. This may explain why many normal BMI individuals (especially Asians) can have high levels of insulin resistance in the liver [75].

Since the liver also controls cholesterol synthesis, insulin resistance in this organ is reflected in growing dysfunction in lipoprotein synthesis. In particular, VLDL particles are increased and HDL levels are decreased [67]. This is easily measured by the TG/HDL ratio that is a good general clinical marker for liver insulin resistance [76].

#### Skeletal muscle

Skeletal muscle represents the key site for glucose uptake. Thus reducing insulin resistance in this organ becomes a primary strategy for managing diabetes. Unlike the adipose tissue where macrophage infiltration is a key indicator of inflammation, there is very little macrophage infiltration observed in skeletal muscle in individuals with insulin resistance [77]. It appears that cytokines coming from other organs (adipose tissue and liver) may have the important impact on the development of insulin resistance in the muscle. However, enhanced signaling through the TLR-4 receptor by saturated fatty acids can reduce fatty acid oxidation of the lipids in the muscle [78]. In addition, palmitic acid is the preferred substrate for ceramide synthesis [79]. Whereas ceramide levels are not related to insulin resistance in the liver, they are strongly related to insulin resistance in the muscle [80]. The skeletal muscle is unique that exercise can overcome insulin resistance in this organ by increasing the oxidation of accumulated fatty acids and enhancing the transport of glucose into the cell [81]. This suggests that the molecular drivers of insulin resistance can be different from organ to organ.

#### Pancreas

Although the beta cells of the pancreas sense glucose levels in the blood (via glucokinase) [82] and secrete insulin in response to those levels, the beta cells of this organ are not normally considered targets of insulin resistance. However, the beta cells are very prone to toxicity mediated by inflammatory agents. In particular, 12-HETE derived from AA is very toxic to the beta cells [83]. With the destruction of the beta cells by 12-HETE, the pancreas is no longer able to maintain compensatory levels of insulin secretion to reduce blood-glucose levels and the development of type-2 diabetes is rapid.

#### Gastrointestinal (GI) tract

Like the pancreas, the GI tract is also not considered a standard target organ for insulin resistance, but it is the first organ in the body for nutrient sensing of molecules that can ultimately affect insulin resistance. This begins in the oral region. Fatty-acid receptors such as GPR120 and GPR40 and fatty binding proteins such as CD36 are present in the mouth and line the entire GI tract [84]. Essentially, these receptors allow for the “tasting” of the fatty acid content of diet. CD36 binds oleic acid and helps convert it into oleylethanolamide (OEA) [85]. OEA activates PPARα gene transcription factor to increase satiety and also the expression of the enzyme required for fatty acid oxidation [86]. Thus the type of fat sensed in mouth and gut provides satiety signals to hypothalamus. The increased satiety lowers the overall caloric intake and reduces development of ER and oxidative stress thus indirectly reducing the development of insulin resistance.

Although the GI tract is a long and complicated organ, the enteroendocrine cells that produce hormones in the GI tract represent less than 1 % of its total cells [84]. These specific cells sense and respond to specific nutrients by secreting more than 20 different hormones [87]. The primary hormones secreted by these cells that relate to insulin resistance include CCK (from the proximal I-cells) and GLP-1 and PYY (from the distal L-cells).

CCK is the hormone secreted from the I-cells in response to the fat content in a meal [88]. This is short-acting hormone and works in association with serotonin to suppress hunger by directly interacting with the hypothalamus via the vagus nerve [89, 90]. In animal models being fed a HFD, the satiety signals of CCK to the hypothalamus can become attenuated probably by increased inflammation in the hypothalamus [91]. CCK can also reduce glucose synthesis in the liver probably through its interaction with the hypothalamus [92], but only if its hormonal signaling pathway is not being disrupted by inflammation within the hypothalamus.

PYY and GLP-1 are the hormones released by protein and glucose respectively when sensed by the L-cells more distal in the GI tract. Both of these hormones are powerful inducers of satiety [93, 94]. It has been shown that PYY responses are lower in obese individuals compared to lean individuals [95]. Animal models that have increased levels of PYY due to transgenetic manipulation are resistant to dietary induced obesity [96]. It should be noted that PYY levels rapidly rise after gastric bypass surgery helping to explain the long-term weight loss success of this surgical intervention [97].

Finally, any mention of the GI tract would not be complete without discussing the microbial composition of the gut. It is known that the microbiota is different in lean and obese individuals [98, 99]. The microbial composition also may be a source of low-grade intestinal inflammation especially via endotoxemia mediated by the lipopolysaccharide (LPS) component of gram-negative bacteria that interacts with the TLR-4 receptor. TNFα is up regulated in the ileum of the GI tract by HFD before weight gain is observed in animal models [100]. It is also known that a single high-fat or high-carbohydrate meal can induce such endotoxemia during the increased permeability of the gut during digestion [101–104]. Thus a diet that is higher in protein and lower in both carbohydrate and fat should reduce endotoxemia. Any LPS fragments that enter the blood stream are carried by chylomicrons to the lymph system where it can then interact with the TLR-4 receptors in the body to increase TNFα levels that can generate insulin resistance in a wide variety of organs [105]. Furthermore, it has been demonstrated in animal models that a high-fat diet can initiate insulin resistance via endotoxemia as well as change the composition of the gut microbiota [106, 107]. It has also been recently demonstrated that composition of the high-fat diet (either rich in saturated fat or omega-3 fats) can dramatically alter the composition of the gut microbiome and influence the levels of endotoxemia in animal models [108].

### Summary

Insulin resistance is easy to define, but complex to understand at the molecular level. The same is true for inflammation. This leads to a major limitation of this review because of the integral relationship of fatty acids to inflammation especially as precursors to eicosanoids as modulators of inflammation. In this more limited review, we have tried to focus on the role of fatty acids interactions with specific binding sites in different organs or their synthesis into non-hormonal lipids that may be related to the wide range of the adverse metabolic consequences associated with insulin resistance.

It appears that insulin resistance starts in the hypothalamus causing a disruption in the balance of satiety and hunger signals. This leads to overconsumption of calories. Although excess calories can be theoretically stored safely in the adipose tissue, as the inflammation increases in this organ and insulin resistance develops in the fat cells, the ability to safely store excess fat is compromised. One of the consequences of insulin resistance in the adipose tissue is that excess fat is released into the blood stream and is sequestered by other organs (liver and skeletal muscles) that are not equipped to safely store this excess fat. This is the start of lipotoxicity. With increased lipotoxicity, the metabolism and energy generation becomes compromised, and the development of chronic diseases (diabetes, heart disease, and polycystic ovary syndrome) associated with insulin resistance becomes accelerated. The levels of fat in the diet and the composition of those fatty acids in the fat component can have a significant role in the modulation of insulin resistance.
